# Supplementary material for: Cooperation Between Rhodococcus qinshengii and Rhodococcus erythropolis for Carbendazim Degradation
Source: Microorganisms. 2024 Dec 29;13(1):40. doi: 10.3390/microorganisms13010040 (PMC11767360; doi:10.3390/microorganisms13010040)
Supplement: Supplementary file 1 [file microorganisms-13-00040-s001.zip › microorganisms-3385831-supplementary.pdf]

Table S1. Enzymes involved in carbendazim degradation in *Rhodococcus* strains.

Enzyme	BLASTP alignment						Accession number	Localization
	R. qingshengii RC1			*R. erythropolis* RC9				
	% Cover	% ID	E value	% Cover	% ID	E value		
Carbendazim hydrolyzing esterase (MheI)	54	34.8	4.00E-13	100	99.6	0	ACV42482.1	Plasmid
Carbendazim hydrolase (CbmA)	100	99.7	0	100	99.8	0	ACV42482.1	Chromosome
Ring hydroxylating dioxygenase alpha subunit *Gordonia* sp. KTR9 hydroxylase (EdoB3)	80	27.365	2.00E-46	90	64.51	0	WP_014925847.1	Plasmid
Acyl-CoA dehydrogenase family protein hydroxylase (Hdx)	100	100	0	100	98.98	0	WP_003944352.1	Chromosome
Acyl-CoA dehydrogenase family protein *Actinomycetes* (Hdx)	100	100	0	100	100	0	WP_003940250.1	Chromosome
Extradiol dioxygenase (EdoA)	100	94.9	1.00E-173	100	90.1	5.00E-167	WP_073512241.1	Chromosome
NAD(P)/FAD-dependent oxidoreductase Sphingomonadales (EdoB1)	98	39.07	1.00E-88	99	45.19	2.00E-100	WP_007015992.1	Plasmid
Glyoxalase/bleomycin resistance protein/dioxygenase *Dechloromonas aromatica* RCB (EdoC)	96	38.49	3.00E-60	98	42.76	1.00E-68	WP_011287093.1	Plasmid

3-phenylpropionate/cinnamic acid dioxygenase subunit beta *Rhodococcus* sp. JVH1 (EdoB2)	95	26.52	9.00E-06	98	59.36	5.00E-76	WP_009478597.1	Plasmid
Monooxygenase (Mno)	100	99.8	0	100	99.8	0	WP_003939685.1	Chromosome
Monooxygenase (Mno)	100	99.08	0	76	34.79	5.00E-99	WP_007735571.1	Chromosome
Monooxygenase (Mno)	100	100	0	100	96.3	0	WP_007735567.1	Chromosome
Benzoate 1,2-dioxygenase large subunit (BenA)	100	99.6	0	100	99.6	0	WP_003940124.1	Chromosome
Benzoate 1,2-dioxygenase small subunit (Ben B)	100	100	2.00E-127	100	100	2.00E-127	WP_003939939.1	Chromosome
catechol 1,2-dioxygenase (CatA)	100	100	0	100	100	0	WP_003940427.1	Chromosome
Muconate/chloromuconate family cycloisomerase [*Rhodococcus*] (CatB)	100	100	0	100	100	0	WP_003940572.1	Chromosome
Muconolactone Delta-isomerase [*Rhodococcus*] (CatC)	100	98.92	2.00E-66	100	98.92	2.00E-66	WP_003940572.1	Chromosome

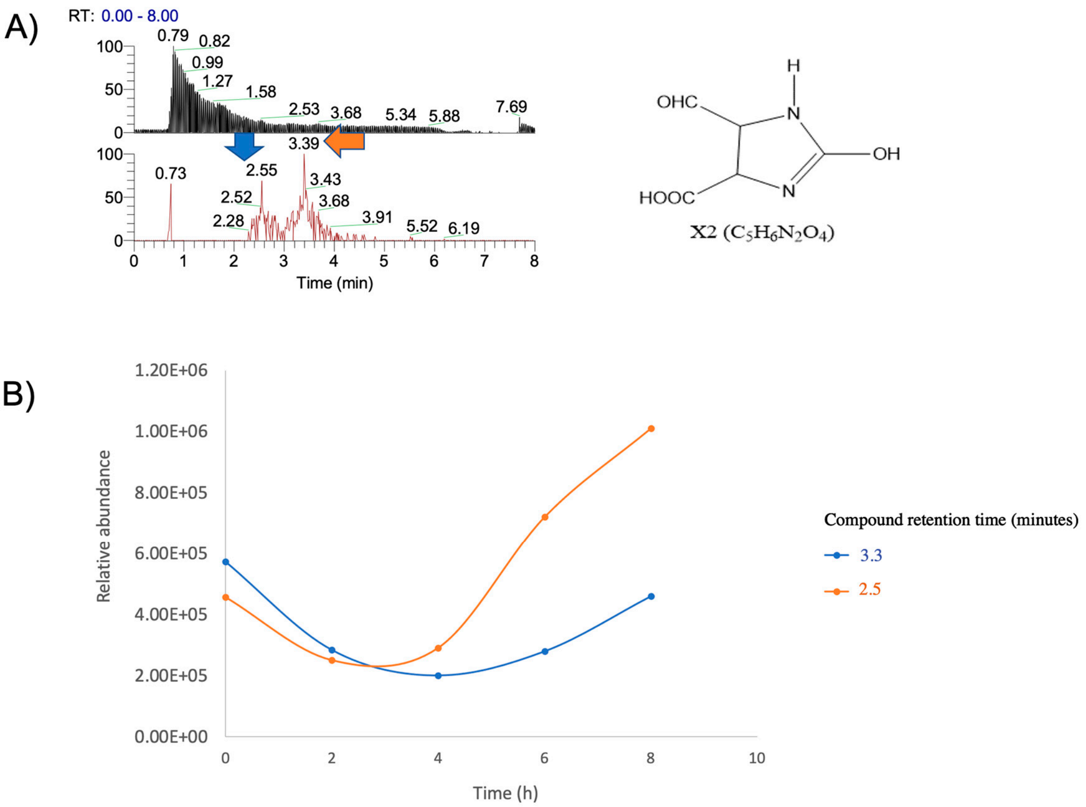

Figure S1. HRESIMS-MS/MS analysis of compounds with *m/z* 159.0395. A) Total ion chromatogram (TIC) in positive mode (ESI+; upper panel) and selective ion extraction (lower panel). B) Relative abundance of *m/z* 159.0395 (2.5 and 3.3 minutes) in time course analysis (8 hours).
